# Measurement of Phase-Contrast MRI mitral flow and lateral wall motion for Assessment of Diastolic Function in a Normal Collective

**DOI:** 10.1186/1532-429X-13-S1-P122

**Published:** 2011-02-02

**Authors:** Liane Kecker, Stephanie Lehrke, Dirk Lossnitzer, Grigorios Korosoglou, Evangelos Giannitsis, Hugo A Katus, Henning Steen

**Affiliations:** 1 University Heidelberg, Heidelberg, Germany

## Introduction

In approx. 50% of cases, heart failure is caused by an isolated diastolic dysfunction (DD) in the presence of a preserved systolic function but with comparably devastating outcome. Among others, echocardiography (EC) categorized DD mainly according to early (E-wave) and late (A-wave) diastolic mitral blood flow (MBF) as well as tissue-doppler imaging (TDI) showing characteristic S`-E`-A` lateral wall velocity patterns.

Cardiovascular magnetic resonance (CMR) has excellent capabilities to assess blood flow and myocardial tissue motion using phase contrast (PC-CMR) imaging but has not been used to quantify DD similar to the EC approach.

## Purpose

We introduce TDI-comparable tissue-phase-contrast imaging (TPCI) of the lateral wall and present reference values for MBF and TPCI in a normal collective.

## Material and methods

In 120 male/female healthy volunteers divided into three age groups (1=20-35ys;2=36-50ys.;3=>51ys) MBF and TPCI was measured by single-slice short axis PC-CMR (60phases, velocity-encoding=100cm/s) comparable to typical EC locations at the tip of mitral leaflets in diastole on a 1.5T whole body MRI system (Philips Achieva). Similar to EC, mitral E-and A-waves, lateral S`-E`-A-velocities, E/A-, E`/A`- and E/E`-ratios were calculated and compared using ANOVA statistics (p<0.05=significant).

## Results

**Figure 1:**
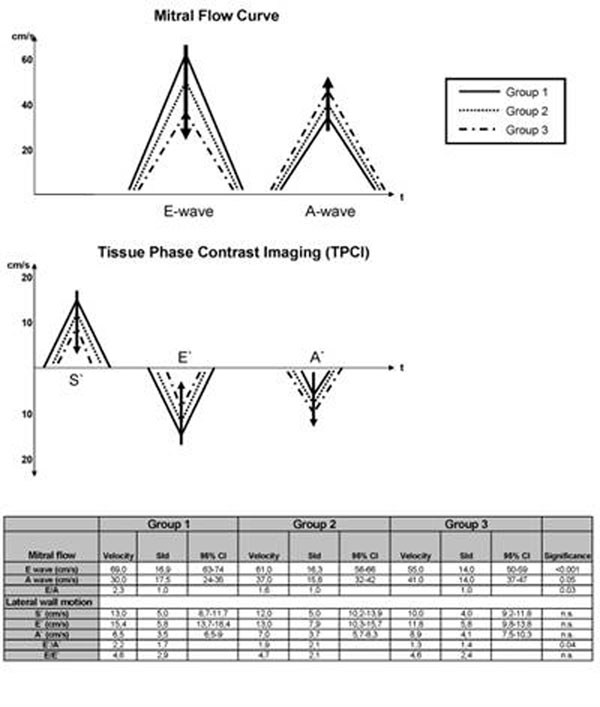


From group 1 to 3, for MBF there is a significant E-wave decrease, A-wave increase and E-A ratio decrease (p<0.05). Similarly albeit not significant for TPCI, we measured an S`-and E`-wave decrease but A`-wave increase. The E`/A` ratio decreased significantly (p=0.04) whereas E/E` remained unchanged for groups 1-3 (figure 1). Mean scan-time was 2.40±1.12 min, mean analysis time was 3.00±1.2min.

## Conclusion

By applying PC-CMR to a normal collective we showed for the first time that TPCI is feasible and can be utilized for evaluation of DD similar to the echocardiography approach in a reasonable scanning and analysis time. The generated reference values for MBF and TPCI could be potentially utilized for future evaluations for DD. However, further studies have to be conducted comparing PC-CMR with conventional EC.

